# A critical review on modulators of Multidrug Resistance Protein 1 in cancer cells

**DOI:** 10.7717/peerj.12594

**Published:** 2022-01-05

**Authors:** Vivian Osei Poku, Surtaj Hussain Iram

**Affiliations:** 1 Department of Chemistry and Biochemistry, South Dakota State University, Brookings, SD, United States of America; 2 American University of Iraq, Sulaimaniya, Sulaimani, KRG, Iraq

**Keywords:** MRP1, ABCC1, ABC transporter, Multidrug Resistance, Anti-cancer agents, MRP1 modulators, Drug absorption and disposition, Drug-transporter interactions, Drug profiling

## Abstract

Multidrug resistance protein 1 (MRP1/ABCC1) is an ATP-dependent efflux transporter, and responsible for the transport of a broad spectrum of xenobiotics, toxins, and physiological substrates across the plasma membrane. As an efflux pump, it plays a significant role in the absorption and disposition of drugs including anticancer drugs, antivirals, antimalarials, and antibiotics and their metabolites across physiological barriers in cells. MRP1 is also known to aid in the regulation of several physiological processes such as redox homeostasis, steroid metabolism, and tissue defense. However, its overexpression has been reported to be a key clinical marker associated with multidrug resistance (MDR) of several types of cancers including lung cancer, childhood neuroblastoma, breast and prostate carcinomas, often resulting in a higher risk of treatment failure and shortened survival rates in cancer patients. Aside MDR, overexpression of MRP1 is also implicated in the development of neurodegenerative and cardiovascular diseases. Due to the cellular importance of MRP1, the identification and biochemical/molecular characterization of modulators of MRP1 activity and expression levels are of key interest to cancer research and beyond. This review primarily aims at highlighting the physiological and pharmacological importance of MRP1, known MRP1 modulators, current challenges encountered, and the potential benefits of conducting further research on the MRP1 transporter.

## Introduction

The advancement of combinatorial chemotherapy regimen to cure cancers has resulted in improved survival rates and quality of life for most cancer patients. Despite the success of this treatment modality, multidrug resistance (MDR) has limited its effectiveness. MDR is a phenomenon in which tumor cells develop resistance to a variety of drugs (Lehnert, 1996). Currently, three mechanisms are known to cause MDR; decreased uptake of water-soluble drugs, cellular changes in cells that reduce the ability of cytotoxic drugs to kill cells, and increased energy-dependent efflux of hydrophobic drugs (Szakács et al., 2006). The pharmacological goal of chemotherapeutic agents is to deliver as much active drug as possible to the molecular target in cancer cells to cause enough cellular damage leading to cell death. However, the reduction in drug accumulation (low dose) is one of the factors that decrease the amount of active drug components reaching tumor cells (Molinski et al., 2017; Osa-Andrews et al., 2018). Recent advancement in clinical research has led to the use of nanoparticles (NPs)-based delivery systems as drug carriers allows controlled drug release from the matrix, improves drug bioavailability as well as provides versatile routes of drug administration (Gelperina et al., 2005). Nonetheless, due to the potential toxicity and insufficient transportation of these NPs, only a few have been accepted for clinical treatment (Yin et al., 2018). Aside these drawbacks, recent studies have revealed that ATP-binding cassette (ABC) transporters may be involved in the efflux and detoxification of some of these NPs thereby decreasing their efficacy (Al-Hajaj et al., 2011; Chen et al., 2016; Tian et al., 2019; Yuan et al., 2021).

The ABC transporters are a superfamily of transporters that were initially identified in studies related to nutrient uptake in bacteria in the 1970s (Berger & Heppel, 1974). P-glycoprotein was the first ABC transporter to be discovered in 1973 (George, 2016). Following subsequent studies, this superfamily of transporters was recognized to be ubiquitous and involved in diverse biochemical and physiological processes (Hyde et al., 1990).

The ATP-binding cassette (ABC) transporters play a pivotal role in the removal of hydrophobic drugs across plasma membranes. Unfortunately, some of these therapeutic agents are substrates of these transporters hence they are effluxed out leading to a decrease in intracellular drug concentration. This goes a long way to affect the bioavailability of the drug as well as their therapeutic impact on tumor cells.

Various proteins in the ABC superfamily are reported to be involved in the absorption, excretion, and distribution of drugs in normal cells (Szakács et al., 2008), however, in cancer cells (where these transporters are overexpressed), therapeutic agents administered are challenged by the efflux activity of these membrane-bound efflux pumps mainly ABC transporters like P-glycoprotein (P-gp) and Multidrug Resistance Protein 1(MRP1) since they serve as the first line of defense (Gottesman, Pastan & Ambudkar, 1996; Kool et al., 1997).

MDR remains a major impediment to the treatment of cancers as its resulting ineffectiveness of the drug treatment is responsible for a larger percentage (about 90%) of cancer-related deaths (Li et al., 2008; Longley & Johnston, 2005; Si et al., 2019), hence the role of ABC transporters which contribute to MDR cannot be overlooked.

## Survey methodology

An extensive survey of existing literature was conducted using Google Scholar and PubMed to analyze the role of ABC transporters in the development of MDR in cancer, with a focus on the MRP1 transporter, its modulators, challenges that limit their efficacy as well as potential strategies that can be explored in overcoming MRP1 mediated MDR in cancer cells. Scientific literature reviewed was not refined by journal type, authors, or publishing date. We also considered results from our previous research works on the MRP1 transporter and cross-referenced published literature to identify other appropriate and related resources.

## Overview of ATP-Binding Cassette Transporters

Membrane transport proteins are one of the essential proteins that aid in the transfer of several classes of molecules in and out of cells. Ion channels, transporters, aquaporins, and ATP- powered pumps are the four different types of membrane transport proteins (Vasiliou, Vasiliou & Nebert, 2009). ATP-powered pumps are ATPases that move ions or small molecules across a membrane *via* active transport (movement of molecules against a concentration or electrochemical gradient) using ATP hydrolysis as the energy source (Lodish et al., 2000b). Examples of ATP powered pumps include the Na^+^/K^+^ ATPase in the plasma membrane which maintains the Na^+^ and K^+^ gradients typically in animal cells, Ca^2+^ATPases that function to pump Ca^2+^ ions out of the cytosol into the external medium or the lumen of the sarcoplasmic reticulum (SR) of muscle cells (Lodish et al., 2000a; Toyoshima, 2009), and ATP binding cassette (ABC) pumps; a large, ubiquitous, and diverse superfamily of transporters. ABC transporters are a type of ATP-binding cassette pumps that are encoded by ABC genes (Verrier et al., 2008), and play a critical role in the transport of a wide class of molecules including sugars, amino acids, peptides, metabolites, xenobiotics, and toxins across both the extra - and intracellular membrane (Broehan et al., 2013; Labbé, Caveney & Donly, 2011). The ABC transporters are the largest protein transporter superfamily present in all organisms and are categorized into importers or exporters depending on the direction of the transport relative to the cytoplasm (Dassa & Bouige, 2001). In prokaryotes, they act as both efflux and influx proteins extruding drugs and toxins out of the cell and transporting nutrients into the cell respectively, however, they can also act solely as efflux transporters in eukaryotes, where they play a key role in protecting cells from toxins (Videira, Reis & Brito, 2014). In plants, ABC transporters aid in the transfer of molecules like lipids, metals among others (Verrier et al., 2008). Structural analysis of ABC transporters reveals an ABC transport core unit that comprises two nucleotide-binding domains (NBDs) and several hydrophobic α-helices forming the membrane-spanning domains (MSDs). The NBDs consist of highly conserved regions or motifs such as the ABC signature motif, the Walker A and Walker B sequences, the H and Q loops (Vasiliou, Vasiliou & Nebert, 2009). The NBDs facilitate ATP hydrolysis to generate energy whereas MSDs on the other hand use the energy generated by the NBDs to catalyze substrate recognition and translocation across the lipid membrane (Vasiliou, Vasiliou & Nebert, 2009).

In humans, 49 ABC genes are currently known and have been grouped into various subfamilies based on their amino acid sequence and protein domains. This classification includes ABCA (12 members), ABCB (11 members), ABCC (13 members), ABCD (4 members), ABCE (1 member), ABCF (3 members), ABCG (5 members) (Auner et al., 2010; Dean, Hamon & Chimini, 2001). ABC transporters are expressed in several organs such as the liver, kidney, adrenal, lungs, intestines, and at various pharmacological sanctuary sites like the blood–brain barrier and the blood-testis barrier (El-Awady et al., 2017), where they are reported to function as channels, receptors, and transporters (Vasiliou, Vasiliou & Nebert, 2009). ABC transporters like P-gp, MRP1, and BCRP have also been reported to play a pivotal role in drug metabolism specifically in phase O and phase III (Dassa & Bouige, 2001). In phase O, they are reported to regulate the entry and extrusion (exit) of drugs before they reach their pharmacological target, resulting in a significant decrease in the intracellular concentration of the drugs, hence affecting their pharmacological efficacy. In phase III, ABC transporters are known to aid in the complete elimination of metabolized molecules. Due to the important role of ABC transporters in drug metabolism, their overexpression has been associated with the multidrug resistance (MDR) phenomenon, a major opponent to the success of the chemotherapeutic regime. Like most essential proteins, studies have revealed that mutation in ABC transporters genes can lead to crucial and recessive disorders including neurological disorders, retinal degeneration, cystic fibrosis among others (Dean, Hamon & Chimini, 2001).

## The ABCC Subfamily

The ABCC subfamily comprises thirteen members. This family of transporters is primarily associated with the export of amphiphilic anions which includes conjugates of lipophilic compounds with glutathione (Keppler, Jedlitschky & Leier, 1998; König et al., 1999). However, recent studies have shown that ABCC6 and ABCC12 are likely not involved in the transport of drugs since their pharmacological and physiological function in cancer chemotherapy remains unclear (Chen & Tiwari, 2011; Sharom et al., 2011). The ABCC subfamily can be categorized into multidrug resistance protein subgroup (MRPs), sulfonylurea receptor subgroup (SURs), and Cystic fibrosis transmembrane conductance regulator (CFTR). The MRP subgroup is made up of nine members, with the SUR subgroup consisting of two members as listed in Table 1.

**Table 1:** Summary of members of the ABCC subfamily.

ABCC subgroup	Symbol	Alternative name	Tissue localization	References
MRPs	ABCC1	MRP1	Ubiquitous (lungs, kidney, placenta, blood–brain barrier)	Flens et al. (1996), Kourti et al. (2007)
	ABCC2	MRP2	Canicular membrane of hepatocytes. Apical membrane of proximal renal tubule endothelial cells	Kool et al. (1997), Sekine, Miyazaki & Endou (2006)
	ABCC3	MRP3	Liver, colon, intestine, adrenal gland	Kool et al. (1997)
	ABCC4	MRP4	Prostate, testis, ovary, intestine, pancreas, lung	Borst et al. (1999), Gottesman & Ambudkar (2001)
	ABCC5	MRP5	Skeletal muscle, brain, heart	McAleer et al. (1999)
	ABCC6	MRP6	Liver, kidney	Gillet, Efferth & Remacle (2007)
	ABCC10	MRP7	Liver, peripheral blood cells, intestines	Bleasby et al. (2006)
	ABCC11	MRP8	Breast, lung, colon, prostate, ovary	Yabuuchi et al. (2001)
	ABCC12	MRP9	Testicular germ cells, sperms	Ono et al. (2007)
SURs	ABCC8	SUR1	Neuronal cells, pancreatic B-cells	Gribble et al. (1998)
	ABCC9	SUR2	SUR 2A - cardiac and skeletal muscle SUR 2B –vascular smooth muscle	Davis-Taber et al. (2000), Isomoto et al. (1996)
	CFTR	ABCC7	Apical membrane of epithelial cells in exocrine glands	Vankeerberghen, Cuppens & Cassiman (2002)
	ABCC13	MRP10	Liver, fetal spleen, colon, placenta, brain, ovary, liver	Yabuuchi et al. (2002)

Members of the MRPs subgroup can also be classified into two main groups based on their predicted topology: the short and long MRPs (Deeley, Westlake & Cole, 2006). The short MRPs are characterized by four domains which include two membrane-spanning domains (MSD1 and MSD2, which are made up of six transmembrane helices), and two nucleotide-binding domains (NBD1 and NBD2). MRPs classified as short proteins include ABCC4/MRP4, ABCC5/MRP5, ABCC11/MRP8, and ABCC12/MRP9 (Chen & Tiwari, 2011). The long MRPs on the other hand are characterized by an extra poorly conserved NH_2_–terminal region and also predicted to have a third membrane-spanning domain in addition to the two other membrane-spanning domains known as the MSD0 (Deeley, Westlake & Cole, 2006). The MSD0 contains five domain helices. Members of this group include ABCC1/MRP1, ABCC2/MRP2, ABCC3/MRP3, ABCC6/MRP6, ABCC10/MRP7(Iram & Cole, 2011; Iram & Cole, 2012).

## Multidrug Resistance Protein 1 (MRP1/ABCC1)

Studies by Cole and her colleagues identified the overexpression of a transporter gene in a multidrug-resistant human lung cancer cell line (H69AR) which did not overexpress P-glycoprotein (P-gp) (a well-studied ABC transporter) (Cole et al., 1992). This transporter was later found to be an ABC efflux pump, known as MRP1 (Cole et al., 1994). MRP1 belongs to the ABCC subfamily of ABC transporters and is encoded by the gene ABCC1. MRP1/ABCC1 is a 1531 amino acid plasma membrane protein with a molecular mass of 190 kDa (Cole et al., 1994). MRP1 is expressed at normal levels in the lungs, kidney, placenta, and heart (Flens et al., 1996; Kourti et al., 2007), with lower expression levels observed in the colon, brain, small intestine, and peripheral blood mononuclear cells (Flens et al., 1996; Jaramillo et al., 2018; Kourti et al., 2007). High expression levels of the transporter are observed in cells at various pharmacological sanctuary sites like the blood–brain barrier, blood-testis barrier, and in the basolateral membrane of polarized cells (Bakos et al., 2000; Lu, Pokharel & Bebawy, 2015) as well as in cells with high proliferative status such as the reactive type II pneumocytes in the alveoli of the lungs (Bréchot et al., 1998). MRP1 as an ATP-dependent efflux transporter plays a major role in transporting broad spectrum substrates. These substrates include organic anions, metalloids (sodium arsenite, potassium antimonite), toxicants (aflatoxin B1, methoxychlor) folic acids, bilirubin, vitamins, glutathione and glucuronide-conjugates of steroids, leukotrienes, and prostaglandins B12 (Cole, 2014; Deeley, Westlake & Cole, 2006; Munoz et al., 2007). Some endobiotics transported by MRP1 include doxorubicin, vincristine, paclitaxel, ritonavir, irinotecan, methotrexate, saquinavir (Munoz et al., 2007). Due to the ability of MRP1 to transport drugs from different drug families irrespective of their molecular target, structure, and mode of action, (Poku, 2021) MRP1 has been reported to regulate the absorption and disposition of drugs as well as their metabolites across cells (Osa-Andrews et al., 2018, Poku, 2021; Takenaka, Itoh & Fujiwara, 2013).

**Figure 1:**
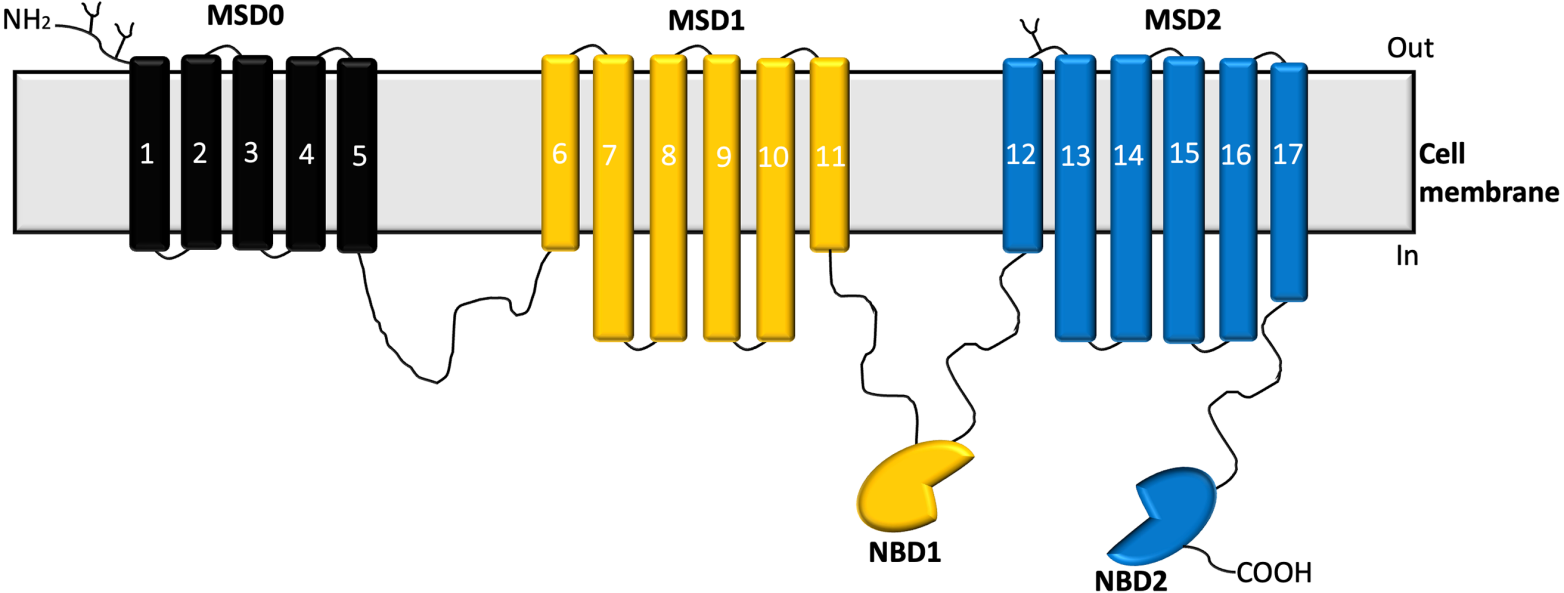
Structure of MRP1 transporter. The membrane-spanning domains; MSD0 (black), MSD1(yellow), MSD2 (blue), and nucleotide-binding domains (NBDs) (Cole, 2014).

Structural analysis of MRP1 reveals two nucleotide-binding domains (NBDs) and two membrane-spanning domains (MSDs) similar to most ABC transporters. The MSDs consist of membrane-spanning domains (MSD1 and MSD2) with each MSD-containing six transmembrane α-helices (Dawson & Locher, 2007). Also, MRP1 possesses a distinct third N-terminal transmembrane spanning domain (MSD0) which comprises five transmembrane spanning helices (Sharom et al., 2011) as shown in Fig. 1.

Studies have shown that MSD0 facilitates interactions between the transporter and other protein partners (Koch et al., 2004). Structural studies have revealed that when MRP1 is not bound to any substrate or ATP, it assumes an inward-facing conformation, while the NBDs are widely separated, and the translocation pathway remains continuous with the cytoplasm (Johnson & Chen, 2017). On the other hand, the MSDs get closer to form a high affinity substrate binding site at which a substrate binds to the transporter, this results in the NBDs moving closer to each other and aligning themselves for dimerization (Johnson & Chen, 2017; Johnson & Chen, 2018). Upon ATP binding, dimerization of the NBDs and rearrangement of the MSDs occurs leading to the outward-facing conformation of the transporter (Johnson & Chen, 2018; Payen et al., 2003). Sequentially, the residues forming the substrate-binding site tend to be pulled apart as the extracellular ends of the helices of the MSDs peel outward leading to a significant reduction in the binding affinity of the substrate to the transporter, the substrate is then released into the extracellular space (Johnson & Chen, 2018).

Recently, several retrospective analyses of chemotherapy results have reported high expression profiles of MRP1 aside from Breast Cancer Resistance Protein (BCRP) and P-gp (Roundhill & Burchill, 2013). Yet, MRP1 is severely understudied although the role of ABC transporters like BRCP and P-gp has been well explored. Interestingly, recent evidence has associated overexpression of MRP1 with a higher incidence of treatment failure, resulting in cancer relapse and poor survival rates in some cancer patients (Greaves et al., 2012). Moreover, the overexpression of MRP1 has also been implicated in the development of multidrug resistance in several cancers like ovarian, lung, breast, pancreatic, kidney carcinomas, and in malignant melanoma (Barrand et al., 1993; Larkin et al., 2004; Li et al., 2009; O’Driscoll et al., 2007; Tong et al., 2019; Walsh et al., 2010; Walsh et al., 2009). Studies by Yang and colleagues also reported elevated expression of MRP1 in colorectal adenocarcinoma and its involvement in the infiltration and metastasis of colorectal adenocarcinoma (Yang, Song & Zhou, 2019). The US food and drug administration (FDA) recommendation in 2017 strongly heralded the need to screen drugs at the clinical trial stages for their likely interaction with MDR proteins such as BCRP and P-gp (FDA, 2020). This recommendation excluded MRP1 even though it is considered as one of the transporters whose overexpression highly influences MDR. Moreover, in addition to anticancer agents, the overexpression of MRP1 has also been reported to reduce the efficacy of various antivirals (saquinavir, zidovudine, ritonavir), antimalarials (berberine), and antibiotics (benzylpenicillin, difloxacin, grepafloxacin) (Deeley & Cole, 2006; Eilers, Roy & Mondal, 2008; Perloff et al., 2001a; Peterson et al., 2017; Shitan et al., 2007; Zhao et al., 2019). MRP1 is also reported to be a major player in the regulation of several physiological processes like redox homeostasis, steroid metabolism, tissue defense, and the etiology of neurodegenerative and cardiovascular diseases (Ballatori et al., 2009a; Ballatori et al., 2009b; Cole, 2014; Krohn et al., 2011; Leslie, Deeley & Cole, 2005; Long, Li & Cui, 2011; Park et al., 2011; Sivils, Gonzalez & Bain, 2010). Considering the unique role of MRP1 and its contribution to MDR, it is expedient to gain more insight into the pharmacological essence of this transporter by profiling for its biochemical interactions with both new and promising drug targets.

### Role of Modulators in Chemotherapy

ABC transporters play a critical role in maintaining normal cellular function and cellular balance. However, cancer cells in their intelligence take advantage of the efflux activity of these transporters by overexpressing these transporters to reduce the antitumor effect of chemotherapeutic drugs. As a result, overexpression of ABC transporters contributes to multidrug resistance in several tumor cells. Studies have shown that ABC transporters like MRP1 play a critical role in the development of multidrug resistance, thus it is of great clinical interest to identify pathways or drugs that can reduce the negative impact due to their overexpression. One feasible approach is to completely shut off these efflux pumps, however, due to their pivotal role in maintaining physiological homeostasis, this option may negatively influence normal cellular balance and function (Sivils, Gonzalez & Bain, 2010; Wijnholds et al., 1997). For instance, studies by Sivils and his colleagues reported reduced testicular steroid hormone levels and alterations in steroid biosynthetic enzymes in MRP1 knock-out mice (Sivils, Gonzalez & Bain, 2010). Likewise, impairment of inflammatory stimulus was observed in mice which had MRP1 knock-out although mice exhibited increased sensitivity to anticancer drugs and were fertile and viable (Wijnholds et al., 1997).

An alternative methodology is to modulate the activity of these transporters in cancer cells *via* biochemical modulation. In this approach, pathways can be biochemically modified by therapeutic agents to enhance the therapeutic potency of anticancer drugs as well as reduce their toxic side effects to normal cells (Fuertes, Alonso & Pérez, 2003; Peters & Van Groeningen, 1991).

Based on the interactions between ABC transporters and various ligands, ligands can be classified as inhibitors, substrates, activators, and inducers. Inhibitors are described as compounds or molecules that either bind directly or indirectly to the transporter to impair its transport activity. Substrates are compounds that are effluxed by the transporter. Activators are compounds that act to enhance the transport activity of these transporters by facilitating conformational changes that promote the transport or efflux of a substrate. Inducers are rather ligands that upregulate the protein expression levels of the transporter (Wessler et al., 2013). By applying biochemical modulation, the activity of the transporter can be inhibited by a modulator without necessarily influencing the normal balance of healthy cells hence, the need to identify modulators is very essential in cancer treatment. Therapeutic agents that inhibit the activity of the transporter could be used together with other anticancer drugs that are known substrates of the transporter. Thus, the identified inhibitor would dampen the efflux activity of the transporter, allowing the anticancer drug to be bioavailable at the appropriate intracellular concentration to exert its effect on the cancer cells. Consequently, enhancing combinatorial drug therapy (use of two or more pharmacologic agents administered separately or in a fixed dose as a single formulation) in cancer patients. Studies on the role of modulators in chemotherapy have also revealed that modulators also possess the side benefit of enhancing oral bioavailability and improving penetration of drugs that are transported by these transporters in tissues (Shukla, Ohnuma & Ambudkar, 2011).

## Modulators of MRP1

MRP1 transporters were discovered several years after the initial characterization of P-gp transporters. Thus, although studies have uncovered and characterized several modulators of P-gp (Luna-Tortós, Fedrowitz & Löscher, 2008; Maitrejean et al., 2000; Starling et al., 1997), there is very little scientific information on modulators of MRP1. For this reason, the goal of identifying modulators of MRP1 is of key interest to oncology research and great clinical value in addressing multidrug resistance. To provide further insight on MRP1 modulators, three main approaches were explored: derivatives of phytochemicals, miRNA-based therapy, and tyrosine kinase inhibitors (TKIs), and other small molecules.

### Phytochemicals and their derivatives

Natural compounds like phytochemicals have been established as compounds that can modulate MRP1 activity. Polyphenols like curcumin, tetrahydroxycurcumin, and bioflavonoids like apigenin, quercetin have been reported to have a significant effect on the transport activity of MRP1 (Li et al., 2010; Morris & Zhang, 2006). Studies have shown that some bioflavonoids can interact with the NBDs of MRP1, hence they can regulate the ability of MRP1 to bind and hydrolyze ATP thereby modulating its transport activity (Morris & Zhang, 2006). Some bioflavonoids are also able to act as competitive substrates whereas others may serve as non-substrate inhibitors. The activities of such natural compounds on MRP1 may be impacted by the presence of glutathione (GSH). For instance, apigenin can enhance MRP1 mediated transport of GSH by six-folds when an increase greater than 10-fold occurs in the apparent Km(GSH) (Leslie, Deeley & Cole, 2003). Other flavonoids like morin, chalcone, silymarin, phloretin, genistein, biochanin A, and kaempferol have also been reported to inhibit MRP1-mediated drug transport (Nguyen, Zhang & Morris, 2003). 3B-Acetyl Tormentic Acid (3ATA), a triterpene isolated from *Cecropia lyratiliba* also inhibits the transport activity of MRP1 by downregulating the total intracellular glutathione (GSH) levels, thereby reducing the activity of glutathione-s-transferase (GST) the enzyme responsible for glutathione conjugation of xenobiotics. Thus, 3ATA was able to sensitize human small cell lung carcinoma cells (GLC4/ADR) to antineoplastic drugs, doxorubicin, and vincristine (Da Graça Rocha et al., 2014). Furthermore, recent studies revealed that the active metabolite of vitamin D3, calcitriol (1,25- dihydroxyvitamin), and its analog, calcipotriol can modulate the activity of MRP1 by specifically inhibiting its transport activity (Tan et al., 2018). Vincristine, a vinca alkaloid isolated from the periwinkle plant (*Catharanthus roseus* (L.) G. Don), used in the treatment of leukemia, lymphoma, rhabdomyosarcoma (soft tissue tumors), neuroblastoma (cancer that forms in nerve tissue) and other carcinomas is also reported to be a well-known substrate of MRP1. Similar activity is also demonstrated by the plant alkaloid and topoisomerase II inhibitor, etoposide (Pommier et al., 2010).

### miRNA and antibody-based therapy

The use of microRNAs (miRNAs) as a therapeutic tool has gained a lot of interest in recent years. It has also gained a lot of attention in the field of oncology where aside from acting as biomarkers for fingerprinting various diseases (circulating miRNAs), they are also reported to play key roles in the development of therapeutic interventions with the added advantage of silencing a wide spectrum of genes (Yeung & Jeang, 2011). Thus, they are reported to have a higher potency in controlling the growth and spread of cancer as compared to the traditional cancer treatment approach. With advancements in miRNA-based therapy for the identification of modulators of MRP1, miR-326 has been reported to down-regulate MRP1 mRNA (Liang et al., 2010). Although the miRNA-based therapies are promising, one major drawback encountered is translating this tool from the bench side to the clinic. Studies have also shown that antibodies like QCRL2, QCRL3 that bind to an MRP1-NBD1 based epitope, and QCRL4 that binds to an MRP1-NBD2 based epitope also exert some inhibitory effect on MRP1 (Cole, 2014; Hipfner et al., 1999). MRP1 Internal Binding 6 (MIB6), an MRP1 specific antibody, inhibited MRP1 ATPase activity in MRP1 overexpressing human small lung cancer cells (Hooijberg et al., 1999a). Recent studies by Li and colleagues also showed that Mab-IR700, an anti-MRP1 antibody exhibit a strong modulatory effect in H69AR cells (Li et al., 2021). However, unlike P-gp where several monoclonal antibodies have been developed against its extracellular epitopes and can inhibit its efflux activity, most MRP1-specific antibodies detect linear epitopes of the transporter but are not able to inhibit its transport activity. This may be because MRP1 is characterized by short extracellular loops, thus little of the transporter is accessible on the cell surface (Cole, 2014). This phenomenon makes developing antibodies targeting extracellular epitopes of MRP1 very challenging and as such, it has been a major hindrance to the antibody-based therapy regime.

### Tyrosine kinase inhibitors and other small molecules

TKIs are small molecules that are designed to inhibit the upregulated activity of various tyrosine kinase receptors involved in cancer (Broekman, Giovannetti & Peters, 2011). TKIs function by hindering the ATP-binding pocket of its tyrosine kinase targets (Cole, 2014). TKIs such as Ibrutinib have been reported to inhibit the activity of MRP1 (Zhang et al., 2014). Rapamycin, an inhibitor of the intracellular serine/threonine kinase (mTOR) that is implicated in the phosphoinositide 3-kinase (PI3K)/Akt signaling pathway, also inhibits the transport activity of MRP1(Brown et al., 1994; Peterson et al., 2017; Sui, Wang & Li, 2008). A Calcein-based high content screening of a unique library of clinically tested anticancer drugs (Z’-factor of 0.63) showed that first-generation rapalogs (analogs of rapamycin)- deforolimus, everolimus, and temsirolimus (ester analog of rapamycin) inhibit the transport activity of MRP1 in small cell lung cancer cells (H69AR), thereby increasing the sensitivity of these cells to vincristine treatment (Peterson et al., 2017). Except for everolimus which had been reported to decrease the MRP1 expression levels in cisplatin-resistant gastric cancer cells (Ying et al., 2014), the effect of other identified inhibitors on the protein expression levels of the MRP1 transporter remains to be elucidated. Other inhibitors that were identified in the study included ESI -09 (a specific inhibitor of EPAC (exchange protein directly activated by Camp), tipifarnib (farnesyltransferase inhibitor), TAK-733 (selective mitogen-activated protein kinase allosteric site inhibitor), HS-173, YM201636 (PI3K inhibitors), AZD1208, CX-6258 (Pan-Pim kinase inhibitors). In another molecular screening conducted in which doxorubicin was used as the fluorescent reporter (average Z’ factor = 0.58) identified several inhibitors (drugs that showed inhibition ≥ 40%) of MRP1 from a unique library of drugs (Sampson et al., 2019). These inhibitors include GSK2126458, MK-2206, mifepristone (progesterone and glucocorticoid hormone antagonist), celecoxib (selective cyclooxygenase inhibitor), and doramapimod (p38 MAPK inhibitor), including two novel inhibitors: alisertib (a second-generation Aurora kinase A and B inhibitor) and amuvatinib (an oral multi-kinase inhibitor of RAD51 and PDGFRa). Other inhibitors identified included flavopiridol, NVP-BSK805, saracatinib, OSI-420, LY294002, rosiglitazone, alvespimycin, LY2228820, GSK461364, GW4064, and afatinib. Other small molecules such as the human immunodeficiency virus type 1 (HIV-1) protease inhibitor, ritonavir has also been reported to induce expression levels of MRP1 in a human intestinal cell line, LS-180 V (Perloff et al., 2001b). Drugs such as rifampicin, dexamethasone, vinblastine, and sulindac have also been reported to be inducers of MRP1 (Nishimura et al., 2008; Schrenk et al., 2001; Tatebe, Sinicrope & Kuo, 2002). Sulindac and its primary metabolites; sulindac sulfide and sulindac sulfone have also been reported to inhibit MRP1 in some studies (Davies & Watson, 1997; Stride, Cole & Deeley, 1999). Substrates of MRP1 include anthracyclines such as doxorubicin, daunorubicin, epirubicin, mitoxantrone, flutamide, and methotrexate (Barrand et al., 1993; Cole et al., 1994; Grant et al., 1994; Hooijberg et al., 1999b; Renes et al., 1999). Cyclosporin A, a known modulator of P-gp has also been reported to modulate MRP1 (Qadir et al., 2005) by inhibiting its efflux activity. Disulfiram, a drug used in the treatment of alcoholism, can also act as a modulator of MRP1 by preventing or inhibiting ATP hydrolysis (Sauna et al., 2004). Structural-activity relationship (SAR) studies of indomethacin mediated MRP1inhibition identified several analogues of indomethacin that inhibited MRP1 activity (Touhey et al., 2002). Likewise, SAR analysis of sulfur-containing verapamil derivatives revealed that the more lipophilic dithiane compounds were more potent in inhibiting MRP1 mediated leukotriene C_4_ (LTC4) transport in the presence of GSH (Loe et al., 2000). Findings from these studies indicate that variations in the molecular structure of current modulators may provide a promising base for the development of potent inhibitors of MRP1. Other inhibitors of MRP1 transport activity include organic acids such as sulfinpyrazone, benzbromarone, probenecid (Hollo et al., 1996), the LTC4 analog MK571 and S-decylglutathione (Eckford & Sharom, 2009; Gekeler et al., 1995). MK571 is one of the standard modulators used in the inhibition of MRP1.

With regards to activators of MRP1, few molecules such as glutathione analogs, specific flavonoids, phenothiazines, some purine, and pyrrolopyrimidines analogs have been reported (Schmitt, 2017; Schmitt, Stefan & Wiese, 2017). This limited number highlights the fact that current knowledge on modulators of MRP1 is still narrow, and therefore calls for more research to be conducted with regards to identifying more potent modulators of MRP1. Surprisingly, most studies investigated the impact of these therapeutic agents on MRP1 activity, but scarcely considered how they affect the protein and gene expression levels of MRP1.

## Conclusion and Future Directions

There is no doubt that the discovery of modulators of MRP1 has had several potential therapeutic benefits especially for patients with drug-resistant tumors. Although most identified modulators of MRP1 have had significant effects on regulating its transport activity, one of the key challenges encountered in clinical trials has been the efficacy and safety of these modulators. Some dreadful side effects and elevated levels of patient toxicities have been reported due to adverse pharmacokinetic interactions with administered anticancer drugs. For instance, the coadministration of cyclosporin A and etoposide to a patient with acute T-lymphocytic leukemia in relapse resulted in progressive hyperbilirubinemia and mental confusion (Kloke & Osieka, 1985). Although toxicity remains a major setback to the success of these modulators, a dose-escalating, single arm, prospective, open label, non-randomized phase I trial of epirubicin in combination with escalating oral doses of sulindac (0–800 mg) in patients with advanced cancer showed that a 600mg oral “pre-dose of sulindac can be combined with a fixed dose of 75mg/m^2^ epirubicin without affecting the conventional toxicity and pharmacokinetics of the anthracycline chemotherapy drug (O’Connor et al., 2007). Moreover, studies by Burkhart and colleagues revealed that reversan, an inhibitor of MRP1 when used in combination with vincristine and etoposide increased tumor sensitivity to these conventional drugs in murine models of neuroblastoma (syngeneic and human xenografts) with no increase in toxicity levels of these conventional agents (Burkhart et al., 2009). Thus, there is the need for further research to be conducted to identify more non-toxic modulators of MRP1 that can be utilized in clinical treatment of MRP1 associated cancers.

Another setback encountered is the genetic variation of single-nucleotide polymorphisms (SNPs). SNPs are defined as polymorphisms that are caused by a point mutation (changes in a DNA base sequence in which a nucleotide may be deleted, added, or substituted) resulting in varying alleles having alternative bases at a precise position of nucleotide within a locus (Jin et al., 2016). The difference in drug absorption, distribution, and elimination has been observed due to single-nucleotide polymorphisms in proteins responsible for drug transport (Kroetz, Yee & Giacomini, 2010). These variations have resulted in differences in toxicity levels and drug response in several patients, including patients with MRP1 SNPs. For instance, MRP1/ABCC1 SNPs in patients with non-Hodgkin’s lymphoma and pediatric cancers resulted in cardiotoxicity in response to anthracycline treatment (Visscher et al., 2012; Wojnowski et al., 2005). Considering the negative impact of SNPs, it would be necessary to adequately genotype clinical trial subjects. Aside from these current challenges, a critical review of literature on modulators of MRP1 reveals that although most MRP1 modulators could influence transporter activity, little is known about their impact on the gene and protein expression levels of the MRP1. Thus, further research must be conducted to investigate how current and future therapeutic agents that interact with MRP1 may affect its protein and gene expression levels. The identification of agents that modulate the expression levels of MRP1 could provide new insights toward the development of more specific and effective therapeutic tools as well as deepen our understanding of the pharmacological and physiological nature of MRP1 transporters.

## Additional Information and Declarations

### Data Availability

The following information was supplied regarding data availability: This is a review article and there is no raw data.
